# Editorial: The Role of Complement in Health and Disease

**DOI:** 10.3389/fimmu.2019.01869

**Published:** 2019-08-07

**Authors:** Maciej Cedzyński, Nicole M. Thielens, Tom Eirik Mollnes, Thomas Vorup-Jensen

**Affiliations:** ^1^Laboratory of Immunobiology of Infections, Institute of Medical Biology, Polish Academy of Sciences, Łódz, Poland; ^2^Univ. Grenoble Alpes, CNRS, CEA, IBS, Grenoble, France; ^3^Department of Immunology, Oslo University Hospital, University of Oslo, Oslo, Norway; ^4^Research Laboratory, Bodø Hospital, K.G. Jebsen TREC, University of Tromsø, Tromsø, Norway; ^5^Centre of Molecular Inflammation Research, Norwegian University of Science and Technology, Trondheim, Norway; ^6^Biophysical Immunology Laboratory, Department of Biomedicine, Aarhus University, Aarhus, Denmark; ^7^Interdisciplinary Nanoscience Center, Aarhus University, Aarhus, Denmark

**Keywords:** complement, classical pathway, alternative pathway, lectin pathway, membrane-attack complex, infection, cancer, renal disease

The complement system is a crucial mediator of the innate immune response, contributing to cell homeostasis, tissue development, and repair, reproduction, and cross-talk with other endogenous systems. Each of the three major complement activation pathways (classical, CP; alternative, AP; and lectin, LP) employs specific recognition molecules and initiating serine proteases. All three pathways converge into a common sequence. This leads to the formation of the biologically highly active anaphylatoxin, C5a, and the C5b-9 membrane attack complex (MAC). The latter forms transmembrane channels which either induces “sub-lytic” activation of the cell, or results in target cell lysis.

Primarily, as an important branch of first-line defense, complement protects the host from invading pathogens and abnormal self-derived components. This can be achieved through several mechanisms: (i) opsonization by activation products, (ii) attracting immune cells through chemotaxis, and (iii) direct lysis after incorporation of the MAC into the cell envelope of the invading pathogen. Therefore, complement plays key roles in (i) preventing the spread of infection to other cells and tissues, (ii) participating in the clearance of damaged cells and tissues, and (iii) preventing the development of chronic inflammation and/or cancer. On the other hand, uncontrolled complement activation may lead to life-threatening effects such as systemic inflammation and shock, dysregulation of coagulation/fibrinolysis, and auto-aggression. Furthermore, under certain conditions, deregulated complement activation may also promote tumorigenesis.

The 49 articles of this issue summarize recent achievements and provide timely reviews in the field of complement research.

## Basic Mechanisms of Complement Physiological and Pathological Functions

The complement cascade needs to be tightly regulated to avoid over activation and inflammatory pathologies. The knowledge on complement regulators has greatly evolved recently, and Sánchez-Corral et al. provided an update on the family of factor H related proteins (FHRs). Their review includes quantitation of circulating FHR proteins, differential interaction of FH and FHR proteins with various ligands, and association of genetic variants and abnormal protein rearrangements with diseases involving renal or ocular pathologies. FHRs are emerging as FH antagonists able to enhance complement activation by outcompeting FH for ligand binding. FH interaction with apolipoprotein E (apoE) has been revisited by Nissilä et al. to decipher its possible role in the anti-atherogenic effects of apoE. FH bound to monocyte cell surface complement receptor (CR3) in the absence of deposited C3b and increased ApoE binding to monocytes and THP-1 cells, possibly through simultaneous interaction with sialic acids. Incubation of FH with THP-1 macrophages and cholesterol-labeled cells enhanced cholesterol efflux and modulated the transcription of inflammatory genes. In addition, binding of FH to THP-1 macrophages decreased complement activation, suggesting an overall contribution of FH apoE interaction in reducing inflammation in atherosclerotic lesions.

Complement receptors provide a connection between soluble complement components and immune cells functions. The review by Vorup-Jensen and Jensen highlights similarities and differences in the structure and function of CR3 and CR4, two members of the family of β2 integrins with similar ectodomain organization. Available X-ray crystal structures and comparison of the wide variety of identified ligands suggest that CR3 and CR4 selectively recognize polycationic and polyanionic species, respectively, with complementary functional implications. The potential role of CR3 and CR4 in human therapy is raised, since existing drugs may target their contribution to both innate and adaptive immunity.

The review by Ratajczak et al. focuses on an emerging role of mannose-binding lectin- (MBL)-dependent complement activation in the release of effector cells from bone marrow (BM) in response to tissue injury, pathogens, and pharmacological inducers of hematopoietic stem/progenitor cells (HSPCs). This process is triggered by MBL binding to ATP, an alarmin released from activated BM cells and contributes to sterile inflammation in the BM microenvironment. ATP is an important signaling molecule, providing an emergent link between purinergic signaling and complement, which participates in homeostasis, mobilization and aging of HSPCs, and possibly in pathologies including myelodysplasia and graft-vs.-host disease in transplanted recipients.

Intracellular C3 activation mediated by cathepsin L (CTSL) plays a central role in the regulation of human T cell responses through signals delivered by C3a and C3b binding to complement receptors C3aR and CD36, respectively. Freeley et al. showed that asparaginyl endopeptidase (AEP), also called legumain, is required for normal Th1 induction and IFN-γ production in both human and mouse CD4^+^ T cells. They also identified AEP as responsible for CTSL processing to an active form, required for CTSL-mediated C3 activation and autocrine CD46 and C3aR activation in human CD4^+^ T cells, but not in mice cells.

This section highlights that complement basic knowledge is continuously emerging with the description of novel functions relying on recently identified ligands of complement components or on cross-talk with different signaling pathways.

## Complement Assays

The review by Ekdahl et al. summarizes the current indications for complement diagnostics and the specific methods used to determine individual complement status, and provides clues to interpret serological complement biomarkers in pathologic conditions. The described assays measure the levels of circulating complement components and activation products, and the function of the different activation pathways, allowing to distinguish between lack of a single component and functional deficiency. The main indications for determination of patient's complement status are deficiencies (inherited and acquired) and pathologies with abnormal complement activation. Additionally serum biomarkers should be useful for the follow-up of patients with complement therapeutics.

Complement biomarkers are especially useful to follow the activity of diseases if they are compared with baseline values under physiological conditions. Gaya da Costa et al. have investigated the serum concentrations of 19 complement components and the activity of the three complement pathways in a healthy Caucasian population of 120 individuals (50% women/men, 20–69 years old). They observed that sex and age significantly impact the levels and functionality of complement, suggesting that these two population parameters should be considered in complement-related pathologies and -targeted therapies.

Mutti et al. addressed the problem of low reproducibility of assays employing normal serum. With the help of affinity chromatography, antibodies may be removed from the serum; however, this procedure depletes serum from complement components associated with CP, LP, and AP. The reconstitution of complement activity (at least its classical pathway) is possible by supplementation with corresponding factors at proper concentrations and in strictly defined order. The proposed method makes possible better standarization which may be helpful in studies involving CP activity.

Clinical tools are still rare for analysis of collectin-11 (CL-11, alias CL-K1), the most recently discovered member of the complement defense collagens family. This protein has been found in the circulation as heteromeric complexes with collectin-10 (CL-10, alias CL-L1) that are able to efficiently activate the lectin complement pathway. Bayarri-Olmos et al. developed a quantitative sandwich ELISA for measuring circulating CL-11, based on two newly generated mAbs. Mean plasma concentrations from 126 Danish blood donors (289.4 ng/ml) were in agreement with the literature and highly correlated to the levels of CL-10/11 complexes. In addition, zymosan was identified as a novel CL-11 ligand able to trigger activation of CL-11/MASP-2 complexes. With a view to understand the mechanism of CL-10/11 complex formation and cellular localization, Hansen et al. studied the tissue distribution of CL-10 and CL-11. Tissues from endocrine organs/glands including liver, kidney, adrenals, and pancreas, as well as exocrine tissues of the digestive system and sex-specific organs showed immunoreactivity for both collectins. The mRNA levels showed accordance between the synthesis site and protein localization. In addition, MASP-3 expression appeared to overlap greatly with the localization of CL-10 and CL-11, which might have functional implications.

The discrimination between normal and abnormal circulating levels of serum components is based on availability of reference intervals (RI) from healthy donors. In the case of FH and FHR-1 to−5, variations in serum levels in adults are associated with multiple diseases. As complement-mediated diseases can have early onset, van Beek et al. assessed the circulating levels of FH and FHR in a population of 110 healthy children (7 months−20.9 years old) and found differences in some, but not all of the RIs of these proteins by comparison with adults. In the case of FHR4, the level of FHR-4A was tested using an assay set-up by Pouw et al. in healthy donor serum samples using novel specific mAbs. These authors showed indeed that FHR-4A is the dominant circulating variant with a level in the range of those reported for all other FHRs whereas FHR-4B could not be detected in serum. In addition, FHR-4A was found to be considerably variable among healthy individuals.

This section illustrates the wide variety of tools available to measure the levels of individual complement components and quantify complement function. The need for standardized tools is highlighted, given the growing number of pathologies involving complement and the development of complement-based therapeutics.

## Therapeutic Tools: Complement Substitution and Inhibition

MBL deficiency is a common condition associated with several pathologies, including increased susceptibility to infections in childhood. Substitution therapies with plasma-derived MBL (pdMBL) proved to be safe with restoration of MBL levels but not of a functional LP due to inactivation of pre-activated MBL-MASPs complexes present in pdMBL. Keizer et al. demonstrated that recombinant MBL (rMBL) can associate with non-complexed circulating forms of proenzyme MASPs and restore LP activity of MBL-deficient serum, providing a rationale for new clinical rMBL substitution studies.

The review by Dobó et al. presents a timely overview of complement therapeutics preventing or inhibiting unwanted/excessive activation of LP and AP. The potential drug targets described encompass LP pattern recognition proteins, serine proteases (MASPs, factors B and D), the central component C3, and complement regulators (factors H and I, properdin). Emphasis is put on recently described cross-talks between LP and AP involving active MASP-3 as the primary physiological activator of pro-FD to FD, and MASP-1 required for AP initiation on LPS containing surfaces. Drug candidates include antibodies, peptides, and selective protease inhibitors for use in the growing number of complement-mediated pathologies.

In a search for new complement inhibitors acting at different levels of the complement cascade, Hertz et al. developed chimeric proteins consisting in MAP-1, a MASP competitor for binding to LP recognition proteins, fused N- or C-terminally to CCP1-5 of C4b-binding protein, a regulator of both CP and LP. Both chimers formed dimers able to associate with MBL or CL-11 while retaining the cofactor activity of C4BP. They could inhibit both CP and LP, exhibiting unique complement-regulatory and anti-inflammatory properties and thus contribute to the emerging field of complement therapeutics.

With a view to develop specific inhibitors of AP and CP C3/C5 convertases, Zwarthoff et al. used C3b-and DNP-coated beads models with purified AP and CP components, respectively, and C3aR and C5aR cell reporter systems to functionally characterize surface-bound reconstituted AP and CP C3/C5 convertases. Screening of therapeutic complement inhibitors and known C3b-binding proteins from human, bacteria or ticks revealed that all molecules tested inhibited C5 conversion in both AP and CP, but only some of them inhibited the C3 convertase. These models open the way to the identification and development of specific convertase inhibitors for treatment of complement-mediated diseases.

## Complement in Infection

Urinary tract infections belong to the most common infectious diseases worldwide. Uropathogenic *Escherichia coli* are aetiological agents of ~80% of cases. Song et al. demonstrated that stimulation of C5aR1-expressing renal tubular epithelial cells (RTEC) with C5a results in elevated expression of mannose residues, depending on the ERK1/2/NF-κB signaling pathway and upregulation of TNF-α production. That facilitates UPEC adhesion and invasion of RTEC, mediated by *E. coli* type 1 fimbriae. Furthermore, higher urine C5a concentrations were found in UTI patients compared with controls. Therefore, it was concluded that C5a/C5aR1 interaction may contribute to the pathogenesis of UTI. Infections, including UTI, are particularly common and often severe in patients suffering from diabetes mellitus. Barkai et al. studied type 2 diabetes mellitus (T2DM) patients with acute bacterial infections, largely UTI caused by *E. coli*. They found higher functional activity of ficolin-3-dependent lectin and alternative pathways, accompanied by lower concentrations of C4d and sC5b-9, in plasma samples, compared with those of non-diabetic patients. The authors suggested generally lower consumption of ficolin-3 dependent LP/AP in T2DM patients resulting in impaired complement-mediated protection from infections. Indeed, lack of ficolin-3-dependent activation and AP amplification were found associated with higher 3-months mortality in diabetic patients.

Another *E. coli* pathotype, EAEC (enteroaggregative) is a worldwide-spread agent of diarrhea. Adler Sørensen et al. reported that ficolin-2 recognized more or less efficiently three of four prototypic EAEC strains but only 5/56 of clinical isolates. The recognition was followed by complement activation, partially inhibitable when ficolin-2 or factor D inhibitors were used and completely blocked when used together. Inhibition of complement activation protected bacteria from direct killing. Therefore, again, although LP activation accompanied by AP amplification is an effective mechanism of elimination of pathogens, many EAEC strains have evolved to evade that. Prototypic EAEC were not recognized by ficolin-1 or ficolin-3 while MBL binding was noted for two strains.

Man-Kupisinska et al. found recognition of enterobacterial lipopolysaccharides by MBL to be common. For most strains, the lectin target was LPS core oligosaccharide. N-acetyl-D-glucosamine and L-glycero-D-mannoheptose were identified as the ligands. They are accessible to MBL *in vivo* when constituting the terminal outer core sugars and the O-polysaccharide is relatively short or when LPS is released due to bacterial cell damage. Using *Hafnia alvei* as a model, it was demonstrated that MBL-LPS interaction not only initiates the complement cascade but also induces early-phase (lipid A-independent) endotoxic shock in mice. Thus, MBL may be involved in life-threatening events in the course of Gram-negative sepsis.

Pertussis (whooping cough), caused by *Bordetella pertussis*, is still one of the most deadly childhood diseases. Hovingh et al. focused on the interaction of Vag8, one of its virulence factors, with C1-inhibitor (C1-INH). That interaction results not only in rescuing bacteria from complement-dependent elimination (as suggested previously) but also in contact system activation. Such an effect was observed when recombinant Vag8 or wild-type *B. pertussis* strain were used but not for the Vag8-knockout bacteria. Sequestration of C1-INH by Vag8 is supposed to hamper its inhibitory activity against βFXIIa and plasma kallikrein. Contact system activation may be additional to the complement evasion mechanism of *B. pertussis* pathogenicity/virulence.

Another major public health problem is tuberculosis. It is estimated that a quarter of the global population is infected with *Mycobacterium tuberculosis*. The lack of sufficiently specific and sensitive biomarkers makes diagnosis difficult as well as differentiation between active and latent infection. Lubbers et al. suggested C1q to be a good candidate TB marker basing on data from several geographically distinct cohorts. The C1q serum concentrations were significantly higher in TB patients, compared not only with healthy controls but also with individuals with latent *M. tuberculosis* infection, leprosy, pneumonia, or sarcoidosis. Moreover, the expression of several complement-associated genes (essentially *C1qB* and *SERPING1*) was upregulated in active TB. Importantly, after half-yearly treatment, C1q levels in the TB group dropped to within the control range. Furthermore, the potential usefulness of that molecule as an active disease marker was supported by data from the non-human primate, *Macaca mulatta*.

Zika virus infections have spread rapidly in recent years. Schiela et al. studied the interaction of Zika virus with complement. The natural (specific against insect components but not against virus) IgM-dependent classical pathway was found to be the major player in complement activation, although direct binding of C1q to NS1 (regulator of viral RNA transcription) and ZIKV envelope were influential as well. Twice diluted serum efficiently reduced number of virions (2 log) while lower (10%) serum concentration had no effect. Based on data from C9-depleted/reconstituted serum, MAC formation was proven to be responsible for viral neutralization.

HIV/AIDS affects nearly 40 million people globally. Coinfections with other pathogens, including HBV and HCV are clinically important, aggravating factors, associated with increased mortality. Tizzot et al. demonstrated that plasma ficolin-1 concentrations were lower in HIV/HCV co-infected patients compared with those infected with HIV alone or controls (the median for controls was in turn lower than for HIV patients). In contrast, average serum ficolin-3 was found to be highest in HIV group and differed significantly from those noted for HIV/HCV patients and healthy subjects. However, ficolin-3 levels within the HIV group correlated with CD4^+^ T cell counts and lower concentrations were found associated with AIDS. Such findings suggest that ficolin-1 and ficolin-3 play different roles in the host response to HIV and/or HCV infections.

*Plasmodium falciparum*, the major agent of another life-threatening disease, malaria (>400 thousand deaths yearly), has developed a variety of pathways to evade host immune responses. Larsen et al. elucidated one of them, associated with *P. falciparum* erythrocyte membrane protein 1 (PfEMP1), expressed on the surface of infected red blood cells. Although recombinant PfEMP1, opsonised by IgG was demonstrated to activate complement *via* the classical pathway efficiently, no activation was observed when native protein was exposed on the surface of erythrocytes. As ability to initiate complement activation depends on antigen orientation, the authors suggested that exposure of PfEMP1 in electron-dense protrusions (knobs) on the cell surface prevents on-target IgG hexamerization and thus consequent C1q binding. Therefore, although antibody response against PfEMP1 is dominated by IgG1 and IgG3 subclasses, it does not lead to complement-dependent lysis of infected erythrocytes.

Other mechanisms of microbial complement evasion were reviewed by Okrój and Potempa. A special attention was paid to *Porphyromonas gingivalis*, the keystone pathogen of periodontitis. *P. gingivalis* expresses outer membrane-anchored proteases, gingipains, able to cleave C5, C3, and C4 components. Moreover, those molecules contribute to the increase of C1 deposition on the cell surface. Interaction of gingipains with C5 results in C5a anaphylatoxin production, generally associated with antimicrobial events. However, C5a suppresses intracellular killing of *P. gingivalis* by macrophages and corrupts the C5aR-TLR-2 cross-talk leading to release of proinflammatory cytokines, contributing thus to bone resorption. Furthermore, gingipains (at higher concentrations) degrade C5b (as well as C3 and C4) which prevents complement-dependent cell lysis. Finally, bacterial peptidyl arginine deiminase (PPAD) is able to citrullinate C5a C-terminal Arg residue leading to loss of its chemotactic activity. Therefore, it is supposed that *P. gingivalis* strategy consists in active controlling (promoting or inhibiting) complement activation at various stages and this “inflammophilic” pathogen utilizes that system to disturb host's homeostasis.

This section includes papers clarifying a variety of microbial-complement interactions, both beneficial and detrimental for the host, and discussion of application of new disease biomarkers and treatment strategies. Last but not least it offers an overview of contemporary methodology for complement research.

## Complement in Cancer

Multiway involvement of the complement system in cancer is widely discussed in the literature. Carcinogenesis is generally associated with changes in the expression of surface antigens, therefore cancer cells may be recognized by the immune system as “abnormal self.” However, numerous reports revealed variety of mechanisms of escape from complement-dependent elimination, utilizing complement system to establish favorable conditions for tumor survival or to metastasis. Such strategies were depicted in two review papers included in this issue. The first one, cited above (by Okrój and Potempa), specifies excessive expression of membrane-bound/production of soluble complement inhibitors, hijacking the latter from plasma by tumor cells and their ability to remove MAC from the surface. Some cancer cells take advantage of local inflammation mediated by anaphylatoxins which may be intensified by endogenous C5 production and C5a generation with their own serine proteases. The detrimental effects of anaphylatoxins, contributing to the proliferation and invasiveness of cancer cells expressing C3a and C5a receptors, was underlined as well in another review, by Kochanek et al. This paper is focused on complement-mediated promotion of cancer metastasis. The invasion-metastasis cascade, leading to spreading of tumor cells through blood or lymph is a complex process involving events in primary tumor, circulation and target sites. It is associated with majority (~90%) of fatal outcomes of disease. The authors delineate contribution of C3a and C5a/C5aR1 to epithelial-to-mesenchymal transition (EMT). Further, the formation of the premetastatic niche (including vascular alterations, activation of resident cells, remodeling of extracellular matrix, and recruitment of immunosuppressive cells) as well as proven experimentally or suspected involvement of complement system in that process are discussed in details. Therefore, targeting some complement factors seem to be promising for development of new life-saving therapeutic strategies.

Two other, experimental papers, concern associations of complement lectin pathway factors with malignancy. Cervical cancer is one of the most common cancers affecting women, evolving from persistent infection with oncogenic types of human papilloma virus (HPV). Maestri et al. found significantly higher serum concentrations of MASP-1, MASP-2, and MAp19 in patients with invasive cervical cancer compared with women diagnosed with low or moderate grade cervical intraepithelial neoplasia (CIN-1, CIN-2, CIN-3). Higher MASP-2 and MAp19 levels were moreover associated with CIN/cancer relapse and deaths. The ROC analysis revealed good sensitivity and specificity of high MASP-2 concentration for worse prognosis of cervical lesions. These data suggest an involvement of afore-mentioned serine proteases and related regulatory protein in the pathogenesis of cervical cancer and/or increase of their concentrations in response to carcinogenesis.

Hematologic malignancies derive from various cells of the immune system. Patients are severely immunocompromised due to disease and therapy what is a reason for high susceptibility to infections and mortality. Swierzko et al. found MBL deficiency to be associated with multiple myeloma while “gt1” genotype (based on analysis *MBL2* gene 3'-UTR) seemed to be protective from lymphomas. Furthermore, serum concentrations of CL-LK (a complex of collectin-10 and collectin-11) were higher in multiple myeloma patients before chemotherapy than in healthy controls. High MBL levels were unexpectedly a risk factor for hospital infections. In contrast, MBL deficiency had no impact, at least during cytopenia. On the other hand, data from follow-up (although from limited number of patients) suggested its association with the most severe infections. Therefore, it was supposed that MBL is fully effective when acts together with phagocytes recovered thanks to autologous stem cell transplantation.

Paroxymal nocturnal hemoglobinuria is caused by a mutation of the *PIG-A* gene, resulting in absence of regulatory CD55 and CD59 on affected cells. It is associated with intravascular hemolysis, thrombosis and bone marrow failure. Previously, it was supposed that the spontaneous remission is relatively common. However, Korkama et al., who extensively investigated Finish cohort of patients, found that disappearance of PNH clone may be related not only to patient's recovery (which occurred to be less common as believed earlier) but also to development of leukemia. Therefore, patients have to be followed-up carefully for a potential of emergence of cancer.

This section demonstrates complex interplay between complement and cancer, from carcinogenesis through metastasis, infectious complications, impact of complement on effectiveness of therapy (and *vice versa—*effect of therapy on complement activity), to positive or fatal outcome.

## Complement in Renal Diseases

The role of complement in the pathophysiology of renal diseases, and particular the potential for treating several of these diseases, have been one of the main focuses of the complement literature the last years. Furthermore, the different triggering events and subsequent pathophysiological development of the renal diseases varies substantially. It is therefore critically important to understand the mechanisms behind the different renal diseases in order to develop optimal treatment regimens for each of them. Some of the diseases definitely are strongly complement driven, whereas other are less dependent on complement, though still complement activation may occur as a secondary process worsening the condition.

In the present issue, nine papers deals with renal diseases including dialysis. The atypical hemolytic uremic syndrome and the C3 glomerulopathies (C3GN), the latter with its two main subtypes, the dense deposits disease (DDD) and the C3 glomerulonephritis (C3GN), are caused by primary dysregulation of the alterative pathway due to mutations in the components. Secondary, destabilization of the alternative convertase may be due to autoantibodies (nephritic factors). In all cases, there are a stabilization or dysregulation of the alternative pathway convertase, leading to enhanced activation of C3 with, in most cases, subsequent C5 activation and destruction of the glomeruli mainly by terminal pathway activation with release of C5a and C5b-9 release. Michels et al. described a novel hemolytic assay to study the alternative convertase in whole serum, implying that both mutations, here exemplified by factor B, and autoantibodies to the convertase can be studied simultaneously. They documented different effects on the convertase by the different conditions and followed the convertase over time in the patients. Thus, they provided a new tool to study the alternative pathway activity in a broad range of renal diseases. The authors postulated targets in the alternative pathway for therapeutic inhibition. The important role of the alternative pathway in renal disease is followed up by Luo et al. who studied the role of the alternative pathway in a mice model of membranous nephropathy, a disease which has been shown to be mechanistically driven by the C5b-9 destruction of the podocytes with subsequent proteinuria. The disease is caused by autoantibodies, mainly of the IgG4 subclass, which does not activate the classical pathway. The role of the lectin and alternative pathway in triggering complement activation is unclear. Interestingly, the authors fond the same amount of subendothelial IgG in wild type as in factor B deficient mice, whereas the latter was protected from proteinuria. Proteinuria was markedly reduced in C5 deficient mice, consistent with C5b-9 being the mediator of the proteinuria. Notably, the authors document that the alternative pathway is crucial for the membrane damage and proteinuria in this model. Future studies will reveal whether this is a direct alternative activation or is due to a strong amplification by a weak triggering signal from another initial pathway.

Different renal diseases with complement-dependent pathophysiology clearly differ with respect to the initial triggering event and the alternative pathway is not necessarily primary, as mentioned above, although it contributes with the amplification mechanism. Limited data are available on the role of the lectin pathway in lupus glomerulonephritis. In a study by Machida et al., it was documented that the lectin pathway molecules MASP-1/3, using knock-out mice for this gene, were essential for development of glomerulonephritis secondary to the lupus-like mouse MRL/lpr model. They concluded that the activation was either by the lectin and/or the alternative pathway. To further illustrate the complexity of initial activation of complement, Chauvet et al. showed that not only the monoclonal antibodies contributes to C3G (MIg-C3), but a number of patients also presented with polyclonal antibodies. This might be of importance for the strategy of treatment of these patients and the authors suggest this as an example of how to design personalized therapy in the future.

Nephritic factors, as mentioned above for the alternative pathway, has for a long time been associated with renal disease. Recent research has revealed several different forms of these autoantibodies, not only intervening with the alternative pathway C3 convertase, but C4 nephritic factors, intervening with the classical pathway, and C5 nephritic factors, which are C3 nephritic factors also stabilizing the C5 convertase, and limited to the C3 convertase, have been described. The mechanism by which the nephritic factors acts has not been fully elucidated. In the study by Donadelli et al., a significant contribution to unraveling the different molecular mechanism of which C3 nephritic factors acts is described.

Another general pathophysiological condition, the ischemia-reperfusion injury, is of importance in kidney damage, e.g., trauma and shock, and not least during kidney transplantation. Castellano et al. showed a mechanism by which ischemia-reperfusion induces trans-differentiation from pericytes to myofibroblasts by regulating the peritubular capillary lumen through pERK signaling. The data from a pig model suggest that C1-inhibitor might be a future treatment option for renal ischemia-reperfusion injury. Notably, C1-INH is not a specific complement inhibitor, but acts potently both in the kallikrein-kinin systems and in hemostasis in general. Since there is a considerable cross-talk between the cascade systems, named thromboinflammation, this is a very interesting approach for the future of attenuating the renal ischemia-reperfusion injury.

For any renal disease, it should be remembered that C3 is locally produced and contributes to the complement activity and pathogenesis in the tissue. Kidney macrophages serve as an important producer of C3, as an additional and important source to the liver derived plasma C3. Here, Liu et al. showed that this local C3 production might lead to the serious condition renal fibrosis via IL-17A secretion, using inhibitors of C3 and C3aR.

Finally, two papers by Poppelaars et al. and Poppelaars et al. focus on the consequences of hemodialysis on complement activation and subsequent undesired effects, not only on the already damaged kidneys, but also on the systemic endothelial and vessel homeostasis. In the first one, the authors timely and importantly reminds us in a review that we should always be aware of the consequences of hemodialysis. The biocompatibility of the devices is of outmost importance. Complement has been used as valuable biomarker for the compatibility, as well as it has been documented that devices that activate complement lead to a series of secondary adverse effects. In the second paper, the authors follow up this reminder with the important notice that intradialytic complement activation precedes the development of cardiovascular events in hemodialysis patients. This is a clinical study supplied with experimental data supporting their conclusions. Both papers highlight the importance of improving biocompatibility of dialysis devices, reducing complement activation, and not least increase the number of donors with reduced damage of their kidneys before, during and after the transplantation. This would shorten the time for patients suffering during dialysis waiting for a kidney graft and a substantially improved quality of life.

## Complement in Other Diseases

Autoinflammation is the activation of the innate immune system to produce a condition where the response damages host tissue by a release of cytokines and other overactivation of immune effector mechanisms. As such, there is a similarity to the autoimmunity of the adaptive immune system except that no major antigenic stimulants seems to be involved. Rather, more elusive initiators are at play, for instance involving underlying mutational defects in signaling pathways, deficiency in certain critical homeostatic mechanisms or, at least for the acute responses, violent inflammatory responses triggered by traumas or other marked insults to the body. Unsurprisingly, the complement system has long been implicated in these events. In the past, however, the contributions of the complement system was mostly described at observational level, while the present volume includes several papers that outlines more clearly mechanistic associations.

Systemic lupus erythematosus (SLE) and rheumatoid arthritis (RA) are usually considered autoimmune diseases. There is a strong association with autoreactive antibodies, which may serve as biomarkers for the disease. Nevertheless, the innate immune system and its autoinflammatory responses are also an important part of the diseases. As reviewed by Holers and Banda, complement activation in RA may happen through the classical pathway and antibodies to collagen, but other means of activations also occur. Importantly, an animal model of RA deficient in C3 does not develop disease and several other modifications of complement activations delay onset of disease. Taken together, there can be little doubt that complement is an important part of RA. In SLE, complement is also activated although the precise mechanisms remain unclear. In consequence, split products of C3 are generated. Troldborg et al. showed that this enables a detection of C3dg as a diagnostic marker for SLE. Apparently, this methodology is superior to measuring C3. As in the case of RA, it implicates complement activation in the disease. These perspectives on SLE, a disease with marked skin manifestations, are further generalized by Giang et al. in a review on complement and skin disease. Indeed, a new wave of attention to complement has occurred after the development pharmaceutically useful complement inhibitors. This could likely alter the treatment of psoriasis and several other skin diseases with the known influences of complement. In psoriasis vulgaris, the most common form of psoriasis, several complement split products can be found in psoriatic lesions and in the skin scales. These inflammatory foci generate enough complement activation products to cause a rise in systemic levels through a “spill-over.” It remains unknown, however, what exact mechanisms are responsible for complement activation in psoriasis.

Ischemic injuries are another setting where the autoinflammatory response acts to enhance morbidity. Nauser et al. explained the role of a relatively recently discovered member of the complement-activating molecules, namely the collectin-11, in renal transplant injury. Here, ischemia upregulates a glycan structure, which becomes a damage-associated molecular pattern, recognized by collectin-11. Yet, our possibilities of reducing such a response still need the elucidation of the precise glycan structure recognized. The endothelium of kidneys may also be target of overactivation of complement during atypical hemolytic uremic syndrome. As shown by May et al., the release of heme in consequence of the hemolysis is correlated to complement-deposition on the microvascular endothelial cells while the macrovascular endothelium is protected by upregulation of heme- and complement-degrading molecules. A similar topic of tissue damage was pursued by Panagiotou et al. with a focus on ischemia in the myocardial injury. The lectin pathway is a contributor to such injury during reperfusion although other means of complement activation also may play a role. Perhaps for this reason, the multifaceted inhibition of complement by C1-inhibitor, which embodies both the MASP as well as C1 proteases, offers protection in myocardial ischemia/reperfusion. However, the authors conclude that there is a lack of high-quality clinical studies of complement inhibition in human acute myocardial infarction. In some ways, the dysregulated activation of complement induced by trauma resemble the ischemic/reperfusion responses with a significant formation of complement split products. However, the review by Chakraborty et al. describes how the inflammatory response in trauma also involves central parts of the adaptive immune system, from antigen presentation to regulatory signaling in both B and T cells. These findings should encourage similar considerations in other setting with dysregulated complement activation.

Organ damage through dysregulated complement activation may also have an origin in infectious diseases. Catarino et al. present evidence of tissue-damaging complement activation in rheumatic fever after infection with *Streptococcus pyogenes*. In this way, ficolins play a double role by both protecting against the infection as well as raising the tissue damage by complement activation also reminiscent of the report on collectin-11.

In asking the question how to handle complement-mediated inflammation in a clinical setting, the study by Orrem et al. provided interesting insight on the relationship between IL-6 inhibition and the expression of the highly inflammatory C5a and C3a receptors. Tocilizumab, a function-blocking antibody to IL-6 receptor, also down-regulates C5a receptor expression in a context of coronary artery disease.

Complement also serves function in physiology outside the usual venues of immunology. Mohlin et al. identified a set of C3 gene variation, which they showed in subsequent expression studies were associated with low cellular secretion or functional defects. Interestingly, these defects may affect functions of this molecule in early phase of pregnancy and in the development of the placenta.

## Author Contributions

MC wrote introduction, Complement in Infection, and Complement in Cancer sections. NT wrote Basic Mechanisms of Complement Physiological and Pathological Functions and Complement Assays sections. TM wrote Complement in Renal Diseases section. TV-J wrote Complement in Other Diseases section.

### Conflict of Interest Statement

The authors declare that the research was conducted in the absence of any commercial or financial relationships that could be construed as a potential conflict of interest.

